# Primary Breast Angiosarcoma: Comparative Transcriptome Analysis

**DOI:** 10.3390/ijms232416032

**Published:** 2022-12-16

**Authors:** Andrés Rincón-Riveros, Jairo De la Peña, Wilson Rubiano, Fabio Olivella, María Martinez-Agüero, Victoria E. Villegas

**Affiliations:** 1 Bioinformatics and Systems Biology Group, Universidad Nacional de Colombia, Bogotá 111221, Colombia; 2 Servicio de Mastología, Hospital Universitario Mayor Méderi, Bogotá 111411, Colombia; 3 Centro de Investigaciones en Microbiología y Biotecnología-UR (CIMBIUR), Facultad de Ciencias Naturales, Universidad del Rosario, Bogotá 111221, Colombia

**Keywords:** primary breast angiosarcoma, breast cancer, differentially expressed genes, transcriptomic studies, vascular neoplasm, Gene Expression Omnibus (GEO)

## Abstract

Primary breast angiosarcoma, with de novo appearance and not associated with exposure to radiation or lymphedema, is a rare pathology representing less than 0.05% of the neoplasms related to this organ. The pathology is characterized by its aggressiveness, poor prognosis, and difficulties in its differential diagnosis. This article reports the case of a 55-year-old white woman with no family history of cancer, with a rapidly growing mass in the left mammary gland that ulcerates and bleeds. It is confirmed as primary breast angiosarcoma by immunostaining in the tumor tissue for CD31, CD34, and FLI-1. In addition, a sample of neoplastic and healthy tissues is collected from the patient for RNA sequencing; the results are contrasted with a tissue sample from a patient with Luminal A subtype of breast cancer, as well as data from other cases of angiosarcoma available in public databases. These findings revealed a genetic profile associated with the immune and inflammatory response in the patient’s sample when compared to available angiosarcoma data; these molecular patterns are consistent with other recent studies. Due to the rarity of the disease, the studies carried out on each patient contribute to the expanding knowledge of the etiology and molecular pathways that are still partially known and continue to be the subject of research. Aside from a comparative transcriptome study, this article aims to provide an update on the state of knowledge about this disease.

## 1. Introduction

Breast angiosarcoma (BA) is a rare tumor of vascular origin, which was first described by Schmidt in 1887 and later by Borrmann in the early 20th century, who reported its high lethality [1]. Breast angiosarcomas can have two origins. The first is spontaneous or de novo, known as primary breast angiosarcoma. It is characterized by a large tumor size, low histological grade, high local recurrence rates, distant metastasis, and lower overall survival [2,3]. Secondary breast angiosarcoma may also result from sequelae of radiation therapy or other treatments prior to breast cancer. Compared with each other, primary angiosarcomas are relatively rare, occur in younger women, and arise from the breast parenchyma, while secondary angiosarcomas occur in elderly female patients, originate in the skin, and infiltrate the subcutaneous tissue and breast parenchyma [4]. It is worth noting that primary and secondary breast angiosarcomas are two biologically different entities [5,6].

The most frequent primary angiosarcomas are of the head and neck, followed by those of the breast. They tend to generate metastases, contrary to secondary angiosarcomas that present a high local relapse [7]. This rare pathology has a great histological variety, mainly represented by fibrosarcomas, myxoid liposarcomas, phylloid tumors, malignant fibrous histiocytomas, and angiosarcomas [8]. Breast angiosarcomas represent about 0.05% of all malignant breast tumors and less than 5% of all soft tissue tumors [9]. It is a rare entity of unknown etiology, occurring mainly in young women between 30 and 40 years old. It is diagnosed in 1 out of 2000 cases of primary breast cancer and has a poor prognosis and high metastasis rates compared with invasive ductal carcinoma [10].

A differential diagnosis should be made by identifying lesions of benign origin, such as abscesses, pyogenic granuloma, or hemangiomas, as well as by identifying malignant vascular lesions, such as lymphangiosarcomas, hemangiopericytoma, intravascular angiomatosis, and Kaposi’s sarcoma [11]. The standard treatment is a surgical intervention with the enlargement of margins for both the primary tumor and metastatic lesions [12].

In studies with a limited number of samples and using immunohistochemistry techniques, some markers of vascular endothelium growth associated with neoplastic tissue have been reported. The results are summarized in Table 1, where the variability of expressions of these markers can be appreciated [3].

Other studies have reported dysregulation in a set of genes. For example, Naka et al. (1998) described that transgenic mice with p53 suppression frequently developed angiosarcoma [16]. Johnson et al. (2007) first reported that galectin-3, a P-galactoside lectin involved in tumor progression, metastasis, and angiogenesis, among other cellular functions, is widely expressed in angiosarcomas [17]. García et al. (2000) described the mutational state of K-ras in angiosarcoma of the heart; later, Weihrauch et al. (2002) also reported K-ras mutations in liver angiosarcoma [18,19]. The ETS-1 transcription factor and proto-oncogene controlled by kinases regulate the expression of metalloproteinases, such as collagenase-1 (MMP-1), stromelysin (MMP-3), and urokinase-type plasminogen activator (uPA); thus, ETS-1 overexpression would favor a tumor progression associated with metalloproteinases MMP-1 [20]. In an extensive study of de novo and secondary angiosarcomas, Styring et al. (2014) found differential expressions in the *MYC*, *RET*, *KIT*, *FLT4*, and *CDKN2C* genes. In that same year, Behjati et al. identified mutations in *PTPRB* and *PLCG1* by sequencing the entire exome of 39 angiosarcomas; these are genes intimately linked to angiogenesis [21]. Table 2 summarizes some of the genes found with alterations in genome or transcriptome studies.

**Figure 1:**
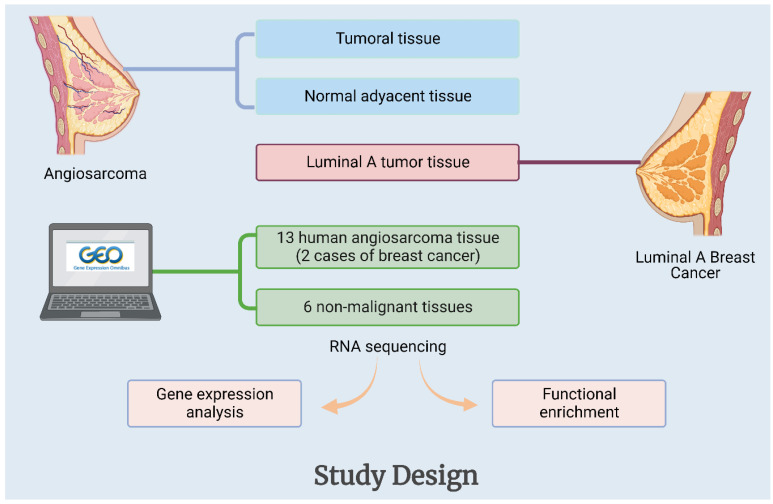
General outline of this study.

This study, in addition to updating what is known about the disease, focuses on the presentation of a case about primary angiosarcoma of the breast, intending to integrate conventional diagnostic information with gene expression analysis. To achieve this aim, the sequencing results of tumor and normal tissue from the same patient are compared with the results of other cases of angiosarcoma in public databases (Figure 1). With permission to use residual tissue from another patient who is being treated at the same hospital for breast cancer, Luminal A subtype of breast cancer, these genetic data were collected and analyzed to determine whether there were common genes that are dysregulated between pathologies. Given the rarity of this pathology, all information collected may help to improve understanding, management, and diagnostic strategies for this disease.

## 2. Results

### 2.1. Case Report

A 55-year-old female patient from Bogotá, Colombia, was admitted to the clinical oncology service of the Hospital Universitario Mayor Méderi, presenting with a rapidly growing mass in the left breast of 3 months of evolution, which subsequently ulcerated and easily bled. A breast ultrasound revealed a solid mass with poorly defined borders, vascularized, and measuring 13 × 6 cm in the upper inner quadrant of the left breast, with an ipsilateral axillary adenomegaly with loss of fatty hilum. It was impossible to perform a mammogram due to the ulceration and bleeding of the mass. As antecedents, she referred to hypermenorrhea, hyperglycemia, osteoarthritis, obesity, sequelae of a fracture in the right femur and left rib cage, and ex-smoking. According to the patient, there was no history of cancer in her family.

**Figure 2:**
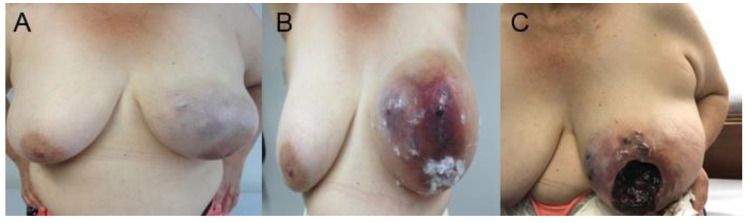
(**A**) An irregular lobulated lesion can be observed that involves 70% of the left breast. (**B**) Progression of the lesion involving the skin and the nipple-areola complex. (**C**) In this tumor type, involvement consists of ulceration and necrosis of the mammary gland.

The initial physical examination showed a large solid mass involving the entire left breast, with skin edema, violaceous satellite nodules, large central ulceration involving the nipple-areola complex (NAC), necrotic edges, active bleeding, and a nodal cluster in the left armpit (Figure 2).

**Figure 3:**
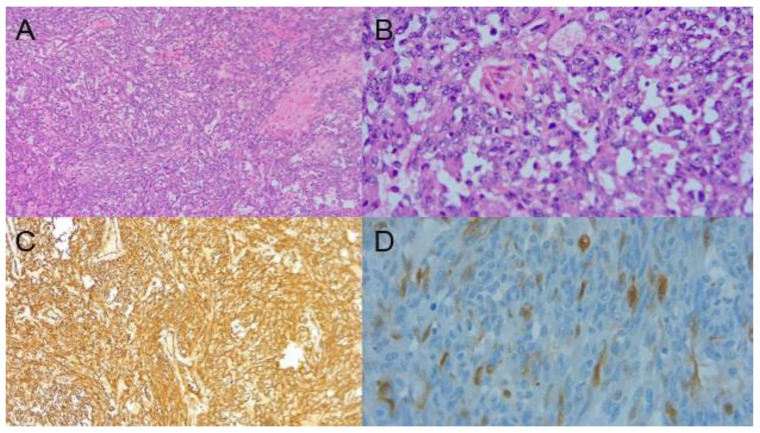
(**A**) H&E 10×; fuso-cellular proliferation with vascular channel formation. (**B**) H&E 40×; higher magnification shows cell population with slight to moderate atypia with vascular channel formation. (**C**) CD34 10×; vascular marker CD34 is diffusely positive in neoplastic cells. (**D**) P16 40×; marker P16 is focally positive in neoplastic cells. H&E = Hematoxylin and Eosin-Stained.

An excisional breast biopsy was performed, with the pathology report revealing an atypical vascular lesion suggestive of angiosarcoma. Immunohistochemistry markers were positive for CD32 and CD34 and negative for CD240, CK 7, high molecular weight CK, and mammaglobin (Figure 3). KI 67 was 40%. The extension images did not evidence distant metastatic involvement.

Due to the characteristics of the mass on physical examination—tumor necrosis and active bleeding that caused secondary anemia and blood transfusion on two occasions—it was decided to perform an urgent modified radical mastectomy.

The surgical pathology report indicated a tumor of 16 × 11 × 9.5 cm with an ulceration of 8.5 × 7.5 cm, malignant cell neoplasm with dermal involvement, negative deep section borders, apocrine metaplasia, and 2 of 18 nodes with macrometastasis. The complementary immunohistochemistry markers were positive for FLI-1 and smooth muscle actin, confirming vascular origin. The other negative markers were HHVB, S100, CK, and BCL-2.

### 2.2. Differential Expression Genes

**Figure 4:**
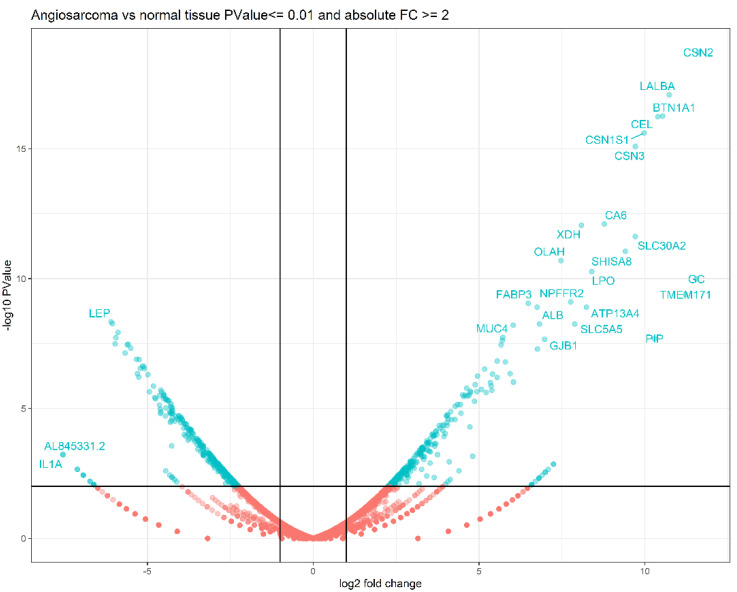
Volcano plot of differential expression of the case report versus adjacent healthy tissue.

The differential expression of the case report was compared to adjacent healthy tissues, resulting in 598 genes with altered expression, where 307 genes were down-regulated and 273 up-regulated (Figure 4).

Regarding the GSE163359 dataset (thirteen human angiosarcoma tissues and six non-malignant tissues), 313 genes showed differential expression: 173 were down-regulated and 140 up-regulated. Table 3 shows the top 20 genes based on adjusted *p*-value < 0.05 and |log2 (FoldChange)| > 2 for each dataset.

### 2.3. Functional Enrichment Analysis

**Figure 5:**
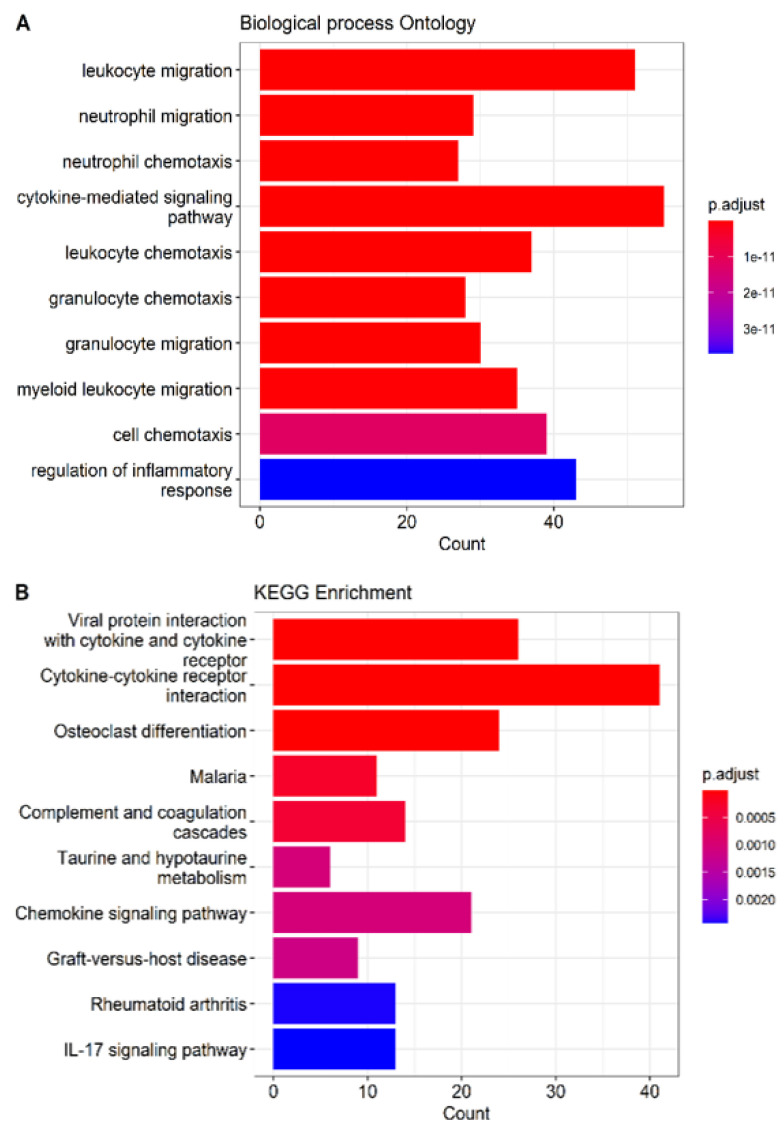
Functional enrichment in differential genes expressed. (**A**) Graphical representation of the GO biological process. (**B**) Metabolic pathways of the KEGG analysis represented in the gene expression signature of the angiosarcoma case reported in this study.

To determine whether the expression profile found in this case report was represented in biological processes and altered metabolic pathways, functional enrichment was performed by applying overrepresentation analysis with Fisher’s exact test, refined with the R package clusterProfiler. The top five of the Gene Ontology (GO) biological processes, with a *p*-value adjusted by Benjamini–Hochberg, are presented in Figure 5. It is noteworthy that the main processes represented in this set of genes are associated with immune response and inflammation, in particular, the mobilization and degranulation of leukocytes. The results of the GO analysis partially agree with what was found in the KEGG analysis, where some routes are also associated with inflammatory processes and immune responses.

### 2.4. Overlapped Genes among Angiosarcoma Case Report and Luminal A Subtype of Breast Cancer Sample

**Figure 6:**
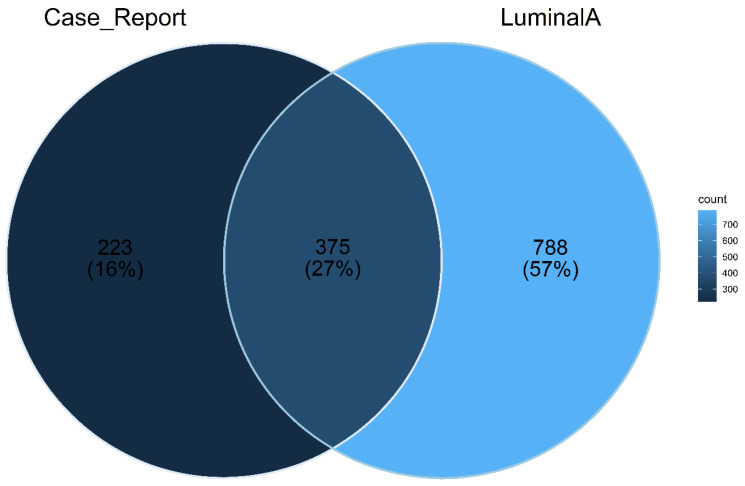
Genes with expression in common between reports of breast angiosarcoma and Luminal A subtype of breast cancer.

It is striking that the genes with the highest variability based on their fold change between the two datasets are inversely expressed. The top five genes overlapping in the expression profiles and their direction are described in Figure 6 and Table 4.

### 2.5. Overlapped Genes between the Case Report and the GSE163359 Dataset

**Figure 7:**
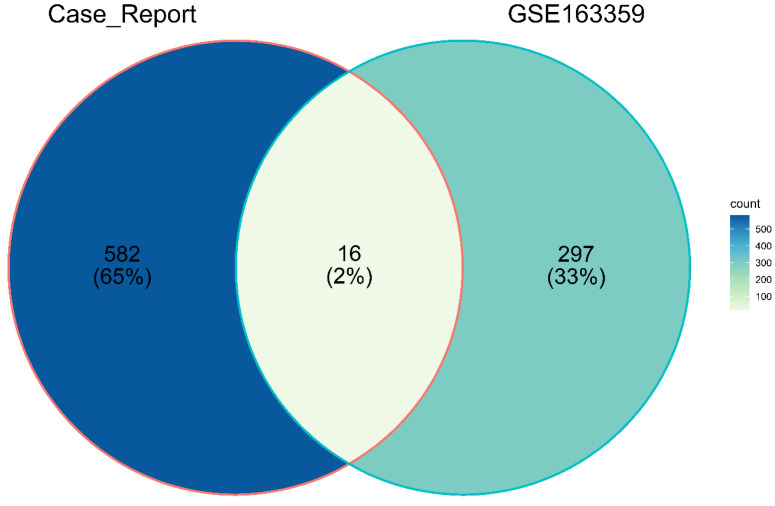
Venn diagram of the genes that overlap in the expression signatures of the two datasets studied.

To establish whether the expression signature found in this case report agrees with genes of altered expression in other human angiosarcomas, the differential expression was compared using a Venn diagram, finding that 16 genes are altered in both datasets (Table 5 and Figure 7). It is worth mentioning that no significant results of differential gene expression were found in the two breast angiosarcoma samples of the GSE163359 dataset and the case study analyzed in this study.

## 3. Discussion

Primary breast angiosarcoma is a pathology characterized by its origin in the vascular endothelium, high aggressiveness at the local level, high rates of distant metastasis, and poor prognosis [11], and it is mostly described in patients between 30 and 40 years of age. It has a high mutational component, with overexpression of genes associated with angiogenesis, such as *TIE1, VEGFR2, SNF-1, TEK* (kinase associated with venous malformations), and *FLT1*. A high expression of markers already known to have an endothelial function, such as PECAM1, EPHA2, ANGPT2, ENDRB, and FLI-1 [26,27], has also been described.

This study reports the pathology in a 55-year-old woman, the age with the lowest number of reports in the literature. Regarding the diagnosis of this case, it was carried out using markers in accordance with those reported in the literature, such as CD32, CD34, and, among others, FLI-1, which played a significant role in the search for an assertive diagnosis. This marker is a transcription factor belonging to the ETS family (E26 transformation-specific), is activated by MAPK, and participates in cell proliferation, differentiation, migration, apoptosis, and metastasis [28,29]. The FLI-1 gene is located on chromosome 11q23, contains nine exons with an extension of 120 kb, and encodes two proteins: p51 and p48. The overexpression of FLI-1 has been associated with some types of leukemia and Ewing’s sarcoma [30,31]. The product of the FLI-1 gene has also been described as a relevant nuclear marker of endothelial differentiation, with an important role in hematopoiesis and angiogenesis, and is very useful in diagnosing pathologies of vascular origin with high sensitivity and specificity >90% [32].

A differential diagnosis in breast angiosarcomas should be made mainly regarding other vascular lesions of the mammary gland, including hemangiomas and angiomatosis, endothelial papillary hyperplasia, sarcomatoid carcinomas, and sarcomas of the breast, where endothelial involvement should be evaluated. Additionally, pseudoangiomatous hyperplasia of the mammary stroma should be ruled out, which presents myofibroblastic proliferation as a characteristic [33].

The treatment of choice in angiosarcoma is radical surgery, considering mainly the size of the mass; conservative surgery is not very effective due to its high rates of local recurrence, although it can be considered in small tumors of a low grade. Treatments with chemotherapy or radiotherapy have uncertain results; no prospective studies have been found that would evaluate the potential benefits of adjuvant chemotherapy [34]. The patient in this case study also underwent a modified radical mastectomy, although she died six months later due to the severity of the disease.

In addition to the clinical–pathological diagnosis of angiosarcoma, a molecular study was performed on the patient. Among the 508 resulting genes with differential expression, the upregulation of three members of the casein family—proteins with effects on tumor cell proliferation—stands out [35]. Alpha-lactalbumin (*LALBA*), a protein associated with mammary gland metabolism that has been found to be increased in tumors of these glands, was also detected to be overexpressed [36]. Another gene that was found to be overexpressed was *HOXA9*, a transcription factor belonging to the HOXA cluster; this group is particularly important in embryonic development and especially HOXA 7–13, which is involved in vascular development [37].

The dysregulated genes with a lower p-adj value were leptin (*LEP*) and cell death-inducing DNA fragmentation factor-like effector (*CIDEC*). Both are involved in lipid metabolic processes and associated with metabolic syndrome and diabetes [38,39]; these have also been reported to be altered in some neoplasms, such as breast and lung cancer.

A recent study published by Wei et al., 2022 [40] reanalyzed two datasets of primary and secondary breast angiosarcomas. Comparing the results of Wei et al., 2022 and the present angiosarcoma case report, 20 genes with differential expression are observed in common: six over-expressed (*PRKAA2, CBS, RAB17, PCSK6, RET, HOXA9, SLC4A11*) and fourteen down-regulated genes (*SORBS1, NRGN, ASPN, DUSP1, GHR, SVEP1, FOSB, TIMP4, ITGAX, TYROBP, FOS, IL1B, PTGS2, NR4A3*). This comparative finding may suggest that there is a common molecular signature between the different types of breast angiosarcoma.

Regarding the comparison of the present case study with the dataset of angiosarcomas from different anatomical locations (GSE163359), altered expressions were found in common in the two datasets as *CFD, ADH1B, ALDH2, MAOA, TMEM132C, GREM2, CLEC3B, PTPRT, CES4A, ADORA1, DLK1, DIO3, ENTPD3, EDN3, C1QTNF7, FRMD1*; these results could indicate a dysregulation metabolic pathway in common among angiosarcomas. In the case of the *DIO3* gene, the alteration of its expression has been reported in other types of sarcomas, such as osteosarcoma and myxoid liposarcoma [41].

A recent study with a significant number of patients with angiosarcoma reported that some patients have a high Tumor Mutational Burden (TMB), which causes a strong immune response, raising the possibility that this characteristic could be a biomarker for the selection of immunotherapy for patients with angiosarcoma [22].

In the enrichment that was carried out with the genes with differential expression in the current case report, several metabolic pathways associated with the immune response were observed, including the migration of leukocytes to the tissues, leukocyte degranulation, and regulation of the inflammatory response. These are processes that are represented in the set of data reported in this work. In this regard, the findings found in the molecular signature are partially aligned with the study by Rosenbaum et al., 2022, who also report an association of the transcriptomic profile in patients with angiosarcoma with inflammatory response and migration of leukocytes to the tumor area [42].

Even keeping in mind that cases of primary angiosarcoma of the breast are different entities from breast cancer, the researchers of this study expected to find dysregulated genes in common in the expression analyses of these two types of tumoral tissues that originate from the same organ. Contrary to expectations, it was found that genes overexpressed in the patient with angiosarcoma were down-regulated in the patient’s sample with breast cancer, specifically Luminal A subtype, and vice versa, a finding that only confirms the differences between neoplasia regardless of whether the affected organ is the same.

## 4. Conclusions

This study found agreement with other findings in the assertive use of markers, primarily CD32, CD34, and FLI-1, in standard angiosarcoma diagnosis; however, it emphasizes the importance of molecular research for genes that express differently among patients, which could cause variations in disease progression and, as a result, affect treatment decisions. The findings presented here, both from the case report and from comparisons with other angiosarcoma datasets, offer an approach to understanding this rare and aggressive tumor, which demands the information to improve diagnosis and decision-making on the best therapy for each patient. It is important to note that the low incidence of this type of relatively uncommon tumor in the population makes molecular characterization difficult, a weakness that can be overcome with comparative studies such as this one, which uses free datasets from diverse populations.

## 5. Materials and Methods

### 5.1. Samples

Fresh tumor tissue and adjacent normal tissue of the patient diagnosed with angiosarcoma were obtained directly from the radical mastectomy performed at the Hospital Universitario Mayor Méderi. A tissue segment was sent for histopathological study, and another was used for RNA extraction and subsequent RNA sequencing. Additionally, tumor tissue from a Luminal A subtype of breast cancer case was processed in the same way to contrast the transcriptome results of these tissues (Figure 1). To summarize, three tissues were contrasted: one malignant and one normal from the same angiosarcoma patient and one Luminal A tumor tissue from a different patient. The samples used in this study had informed consent approved by the Ethics Committee of the Universidad del Rosario.

### 5.2. High-Throughput Gene Expression Data Retrieval

A dataset was obtained from the Gene Expression Omnibus (GEO) http://www.ncbi.nlm.nih.gov/geo/ (accessed on 30 April 2022). The access number of this dataset is GSE163359, and it consisted of 13 human angiosarcoma tissues (including 2 cases of breast cancer) and 6 non-malignant tissues as non-tumor controls (Figure 1).

### 5.3. RNA Isolation

The RNA extraction from tumoral and normal breast tissues was performed with the RNeasy Mini kit (Qiagen, Hamburg, Germany), according to the manufacturer’s instructions. Subsequently, concentration, purity, and integrity were verified using a spectrophotometer, measuring the absorbance at 260/280 nm and electrophoresis under denaturing conditions using formaldehyde. The purified RNA samples were stored at −80 °C until further analysis. Before sequencing, Novogene also evaluated the quality of RNA samples using NanoDrop, agarose gel electrophoresis, and the Agilent 2100 BioAnalyzer.

### 5.4. Angiosarcoma and Breast Cancer Tissues Whole Transcriptome Sequencing and GO-KEGG Enrichment

The RNA library setup and transcriptome sequencing were provided by Novogene Co (Beijing, China). RNA-seq libraries were sequenced as 150-bp paired-end reads using the Illumina HiSeq 4000 platform. The reads were mapped and annotated to the reference genome (GRCh37/hg19) with the STAR software [43]. The differential expression was assessed using Limma and edgeR packages [44,45]. Genes with an adjusted *p*-value < 0.05 and |log2 (FoldChange)| > 2 were considered differentially expressed. For functional enrichment, the R package clusterProfiler was used [46].

## Figures and Tables

**Table 1:** Immunohistochemical reports of the principal markers of vascular endothelium growth found in patients with angiosarcoma and other pathologies related to their respective variability of expression.

References	Sample	Angiogenic Markers in Patients	Additional Notes
Itakura et al., 2008 [13]	34 angiosarcomas	32 (94%) VEGF-A positive. 4 (12%) VEGF-C positive. 32 (94%) VEGFR-1 positive. 22 (65%) VEGFR-2 positive. 27 (79%) VEGFR-3 positive.	VEGF-A expression correlates with VEGFR-1 expression. Loss of VEGFR-2 expression is associated with poor prognosis. VEGFR-1 and VEGFR-3 were not associated with prognosis.
Dim et al., 2007 [14]	20 angiosarcomas 5 hemangioma	All angiosarcomas were VEGF-A positive.	
Zietz et al., 2005 [15]	19 angiosarcomas 10 benign vascular lesions	18 angiosarcomas (95%) VEGF-A positive. 1 benign vascular lesion (10%) VEGF-A positive.	Abnormal MDM2 expression in 68% of angiosarcomas, abnormal P53 expression in 53%; abnormal P53 and MDM2 expression correlates with VEGF expression, which correlates with tumor grade.

**Table 2:** Results of genomic and transcriptomic studies in different types of angiosarcomas.

Methodology	Origin	Genes Altered	Additional Notes	References
Whole-exome sequencing (GES)	Primary breast, cardiac, skin, bladder, lung and others	*TP53*, *KDR*, *PIK3CA*, *POT1*, *PLCG1*, *NF1*, *FAT1*, *LRP1B*, *FLT4*, *NOTCH2*, *PTPRB*, *GRIN2A*, *MYC*	The leading mutated gene in primary breast angiosarcoma was *PIK3CA*.	Painter, C.A. et al. [22]
Multiomic- sequencing and immuno-oncology profiling	Head and neck, breast, liver, lung, and others	*AURKA*, *AURKB*, *PLK1*, *PLK4*, *CHEK1*, and *CDK4.*	TMB was determined high, and there was significant tumor infiltration by immune system cells.	Chan, J.Y. et al. [23]
Retrospective analysis (WES, NGS)	Head and neck, breast, víscera, skin and extremities	*TP53*, *MYC*, *ARID1A*, *POT1*, *ATRX*, *HRAS*, *PIK3CA*	The main changes in breast angiosarcoma were observed in genes such as *MYC*, *HRAS*, and *PIK3CA*.	Espejo-Freire. et al. [24]
Whole-exome sequencing (GES) and RNAseq	Breast, head and neck, chest, víscera, trunk and others	*CIC*, *ETV1*, *ETV4*, *ETV5*, *PLCG1*, *KDR*, *MYC*, *FLT4*	In 50% of angiosarcoma cases, *CIC* was reported to be altered.	Huang SC et al. [25]

**Table 3:** The top 20 genes of the differential expression signature found for: angiosarcoma case report compared to adjacent healthy tissue and the public dataset that corresponded to 13 human angiosarcoma tissues and 6 non-malignant tissues used as non-tumor controls.

Experiment	Up Regulated Genes	Down Regulated Genes
Angiosarcoma Case Report	*CSN2*, *LALBA*, *BTN1A1* *CEL*, *CSN1S1*, *CSN3* *CA6*, *XDH*, *SLC30A2* *SHISA8*, *OLAH*, *LPO* *GC*, *TMEM171*, *NPFFR2* *FABP3*, *ATP13A4*, *ALB*	*LEP*, *CIDEC*
GSE163359	*FSCN1*, *MAP4K4*, *TIE1*, *SHC1*, *MARCKSL1*, *RHOJ*, *SMAD1*, *PPM1F*, *NID1*, *TSPAN18*, *FKBP10*, *ADAM19*, *NAV1*, *DBN1*	*SCARA5*, *DHCR24*, *ZDHHC11B*, *HLF*, *ABCA8*, *IFNLR1*

**Table 4:** Genes with inverted expression (logFC) between the case of angiosarcoma and a case of Luminal A-breast cancer. The results indicate how the same genes over-expressed in an angiosarcoma case are under-expressed in lumina A breast cancer.

Overlapped Genes	Angiosarcoma	Breast Cancer
*CSN2*	11.61	−11.29
*LALBA*	10.73	−11.30
*CSN1S1*	9.97	−5.28
*CSN3*	9.71	−5.73

**Table 5:** Differentially expressed genes in both the case report and the GSE163359 dataset.

Comparison	Overlapped Genes	Count
Case report vs different human angiosarcoma dataset	*CFD*, *ADH1B*, *ALDH2*, *MAOA*, *TMEM132C*, *GREM2*, *CLEC3B*, *PTPRT*, *CES4A*, *ADORA1*, *DLK1*, *DIO3*, *ENTPD3*, *EDN3*, *C1QTNF7*, *FRMD1*	16

## Data Availability

Available RNAseq datasets related to angiosarcoma were downloaded from the GEO repository on the website https://www.ncbi.nlm.nih.gov/geo/.
